# Optimization Design of Street Public Space Layout on Account of Internet of Things and Deep Learning

**DOI:** 10.1155/2022/7274525

**Published:** 2022-08-21

**Authors:** Shanshan Yu, Hao Wang, Yajun Wang

**Affiliations:** ^1^School of Landscape Architecture, Nanjing Forestry University, Nanjing 210037, China; ^2^School of Architecture and Civil Engineering, Xiamen University, Xiamen 361005, China; ^3^School of Architecture and Urban Planning, Fuzhou University, Fuzhou 350116, China

## Abstract

With the gradual improvement of material living standards, people have higher and higher requirements for the livability of modern cities. As an important component of urban construction, the optimal layout of street public space has gradually received more and more attention. In the development stage of the new era, it is very important to improve the image of the city by transforming the street construction, optimizing the urban public space, and building a place full of vitality. Implementing the people-oriented connotation and improving the green travel components in the city, such as encouraging walking and increasing bicycles, are of great significance for optimizing the street public space. This article studies the relevant content of the optimization design of street public space layout based on the Internet of Things and deep learning and expounds the solutions for the optimization design of street public space layout based on the Internet of Things and deep learning. Design research provides cutting-edge scientific theories and evidence. This paper uses data to prove that based on the Internet of Things and deep learning technology, the optimized design of street public space layout has increased the latter's recognition among residents by an average of 21.7%. The designed model has both space utilization and environmental protection. Very good results have been obtained.

## 1. Introduction

Streets are an integral part of every city. In the contemporary society, the street has changed from a pure transportation medium to a multifunctional direction, which requires us to continuously improve the optimization of the street public space. Urban renovation is limited by the traditional model and affected by the car standard, the street experience is reduced, the vitality is limited, and the vitality is lost. These are many abnormal phenomena that appear in the streets. In the development stage of the new era, transforming street construction, optimizing urban public space, and building a place full of vitality are important ways to improve the image of urban streets. One of the manifestations of this new phase of urban development is the improvement of street design, the improvement of urban space levels, and the improvement of the vitality of public real estate. These are the important tasks of urban planning in the new stage of urban development. The research on the optimization design of street public space layout based on the Internet of Things and deep learning provides an excellent reference scheme for solving the above problems, which greatly alleviates the predicament of the original city in terms of space design.

In recent years, the concept of the Internet of Things and deep learning has been widely introduced into many fields. In foreign research, Aswad et al. proposed an Internet of Things-based Flood State Prediction (IoT-FSP) model for river flood state prediction. The IoT-FSP model adopts the IoT architecture for flood data collection and uses three machine learning algorithms, decision tree (DT), decision jungle (Decision Jungle), and random forest (Random Forest) for flood prediction [1]. Lin proposed an automated single-cell electroporation system based on a deep learning algorithm that can automatically detect cells with artificial intelligence (AI) software and deliver exogenous cargoes of different sizes with uniform doses [2]. Anciaes and Jones address the problems arising from the redevelopment of London's neighbourhoods and propose strategies for changing the layout of the local street network and redesigning busy roads [3]. In the domestic research, the research results of the street space layout are remarkable. Some scholars have comprehensively summarized the current situation of road optimization work in the region in their research, sorted out the working principles and management methods issued by the urban planning department in the study area in this field, and discussed the problems existing in practical work. [4]. Starting from the research on the psychological needs of urban residents, some scholars have mastered the local residents' satisfaction with the street layout and the direction of optimization through the questionnaire survey method [5–7]. Based on these survey data, the weight index of the street space optimization design is constructed, so as to quantify and assign points to the existing problems, which provides a useful reference for the development of this research. In addition, among the existing research results, the concept of shared streets proposed by some scholars is also very noteworthy [8–11]. They analyzed the deficiencies in space functions and street optimization of many large cities in China at this stage and then put forward improvement suggestions based on the government's idea of building open communities [12–15]. It is proposed that the closed state of the old community should be gradually broken according to the actual needs so as to realize the optimization and overall renewal of the urban traffic network [16–18].

By analyzing and sorting out the above-mentioned main research results, this research formulates a research framework with the Internet of Things and deep learning as the core of the algorithm. The important components of road layout optimization are analyzed, people-oriented connotation is implemented, and the components of urban green travel are improved [19–22]. While improving the convenience of healthy transportation methods such as walking and cycling, reducing the comprehensive cost of these travel methods is of great significance to the overall image of the city. The research on the optimization design of street public space layout based on the Internet of Things and deep learning proposed in this paper is of great significance for promoting the optimization design of street public space layout, which will inevitably improve the overall layout optimization level of streets and bring benefits to street citizens.

## 2. Design Exploration of Optimization of Street Public Space Layout on Account of Internet of Things and Deep Learning

Internet of Things and deep learning are very popular research methods at this stage, and they have a significant role in promoting various topics.

### 2.1. IoT and Deep Learning

#### 2.1.1. The Internet of Things (IoT)

**Figure 1:**
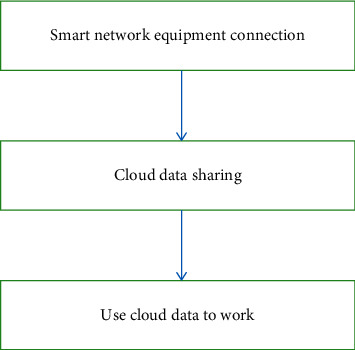
IoT workflow.

The Internet of Things (IoT) is the use of various sensors to link and monitor various objects involved in work in real time [23]. In theory, anything can be included in the IoT system. IoT Workflow is shown in Figure 1.

It works as follows:

1. *Smart Network Equipment Connection*. The component part of the Internet of Things is usually the intelligent part of the network, which uses chips, sensors, and various hardware to extract data from the environment.
2. *Cloud Data Sharing*. IoT devices, various gateways, and checkpoints share the collected data through wireless devices, and the data will be uploaded to the cloud or locally for analysis [24, 25].
3. *Use Cloud Data to Work*. Devices such as Web devices, IoT programs, and AI smart receivers collect and share data, allowing data to be interconnected and set up and accessed [26–30]. Moreover, with the help of information terminals such as artificial intelligence, the Internet of Things can also realize intelligent analysis of the collected data and make accurate predictions on the development trend of things [31–33].

#### 2.1.2. Deep Learning

Deep learning refers to a class of learning algorithms, which is a new class, and these algorithms have been fully used in data applications. A deep learning algorithm is a type of mathematical model that includes multiple processing layers [34, 35]. This data model is trained on a huge amount of data to extract features, so as to continuously improve the recognition, operation, and prediction capabilities of the machine model. Deep learning is different from general machine learning in that this algorithm can learn to recognize objects or objects at a deeper level [36, 37]. Therefore, the data model can mine deeper values in the training data and further improve the performance of the data model by sorting and analyzing the data:

1. Data representation based on multimode
2. Multimodal learning model must meet two requirements: (1) spatial similarity hides relative concept similarity; (2) available for models without models
3. Data representation based on tensors
4. The basic principle of tensor-based data is to connect and display heterogeneous data in a unified way, which means to model higher-order relations of data. The flow is first from video, audio,… XML and HTML data are converted into data streams, and then data flow is quantized, followed by data dimension reduction, data analysis, and finally data service.

### 2.2. Optimization Design of Street Public Space Layout Based on Internet of Things and Deep Learning

Use Internet of Things technology and deep learning algorithms to conduct research on the optimization design of street public space layout, collect street public space data through deep learning algorithms, including street pictures, videos, pedestrian data patterns, traffic flow, buildings and related objects, audio, video, XML documents, GPS data, etc.. Through data stream processing on the collected data, the data are converted into a unified stream, then the data stream is tensorized, and then through dimensionality reduction processing and data analysis, finally, a data service is formed, that is, a street public space layout plan [38, 39]:

1. “Narrow road and dense network” planning mode. It is necessary to make full use of the existing road network and build a road system of “narrow road and dense network.” A dedicated road network for pedestrians and bicycles will be formed to relieve the traffic pressure on the main road and speed up the circulation of vehicles on the main road. At the same time, it also improves the safety of transportation modes such as walking and cycling through the diversion of transportation [40, 41].
2. Reduce the corner radius of the intersection. The study found that the turning radius of road intersections is proportional to the number of traffic accidents. Therefore, when designing a road intersection, the traffic flow and safety should be fully demonstrated. It is not only necessary to achieve the convenience of traffic but also to slow down the speed of the vehicle and increase the safety factor by controlling the angle of the corner.

### 2.3. Algorithm Determination

The research on optimal design of street public space layout uses the Internet of Things technology and deep learning algorithm to intercept street public space data sets and perform the following steps on these basic data:

(1) Data model based on tensors
The basic characteristics of data include time, space, and data service recipients. Therefore, the formula of the tensor is
  (1) $$\begin{array}{c} \xi \in H^{\alpha_{t}\times \alpha_{s}\times \alpha_{u}\times \alpha_{l}\times \cdots \times \alpha_{p}}. \end{array}$$
(2) Tensor expansion operation
Assuming *μ* ∈ *H*^*α*_*t*_×*α*_*s*_×*α*_*u*_×*α*_*l*_^ and *λ* ∈ *H*^*α*_*t*_×*α*_*s*_×*α*_*u*_×*α*_2_^, then the tensor expansion operation is
  (2) $$\begin{array}{c} f=\mu \overset{⟶}{\times }\lambda ⟶\rho ,\rho^{\alpha_{t}\times \alpha_{s}\times \alpha_{u}\times \alpha_{1}\times \alpha_{2}}. \end{array}$$

## 3. Investigation and Research Analysis of Optimization Design of Street Public Space Layout on Account of Internet of Things and Deep Learning

According to research needs, with the support of the Geographic Information System (GIS) technology and geospatial database management technology, team members quantified the spatial layout of streets in each part of the city and integrated them into a unified database. The traffic flow data of the observed intersections are obtained through communication, and then the scientific ratio between ensuring traffic and improving safety is obtained through algorithms, providing systematic and reliable basic data support for the optimization of urban street public space.

### 3.1. The Main Problems in the Layout of Street Public Space

#### 3.1.1. The Phenomenon of Disorderly Parking Is Prominent

**Figure 2:**
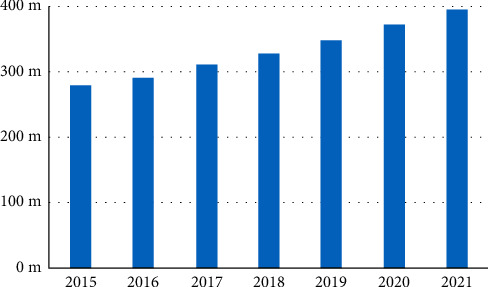
China's motor vehicle ownership from 2015 to 2021.

At this stage, the increasingly prominent problem of urban street space layout is mainly due to the rapid expansion of the number of private cars. With the great progress of domestic automobile technology and the steady improvement of the living standards of urban residents, the car that used to be a symbol of wealthy people in the past has gradually entered the family of ordinary people. Especially after 2015, the number of cars in China's cities has increased rapidly. Data show that, by the end of 2021, the number of motor vehicles in China has reached nearly 400 million, and the per capita ownership rate in some first-tier cities is even more alarming. China's motor vehicle ownership from 2015 to 2021 is shown in Figure 2.

The huge number of private cars not only facilitates the lives of residents but also brings unbearable pressure to the existing road network of the city. The planning and construction of the road network is far behind the growth rate of private cars, and it has become the core problem faced by the optimization of street layout in large cities. Through the analysis of the Internet of Things and deep learning technology, it can be found that many congestions are caused by some car owners parking indiscriminately. The data show that the problem of random parking has caused the average speed of urban arterial roads to decrease by 26.4%, while the incidence of traffic accidents has increased by 28.4% (Table 1).

#### 3.1.2. Street Advertisements Are Chaotic and Low Quality

Throughout the cities of China, the streetscape is almost occupied by various types of advertisements. Bus stops and fences on pedestrian streets are full of commercial advertisements, and advertisements of various businesses continuously bomb pedestrians through high-power loudspeakers. In the process of walking, even people who are walking cannot enjoy the spiritual pleasure. This not only destroys the mood of pedestrians but also creates a lot of safety hazards. At the same time, many cities lack uniform standards for the style management of billboards. In order to attract the attention of pedestrians, many businesses arbitrarily choose the size and color of billboards, which not only reduces the overall aesthetics of urban streets but also increases the degree of danger. In addition, the materials selected by the business billboards are also very different, causing a certain amount of environmental pollution.

### 3.2. Optimization of the Street Public Space Layout Model Based on IoT and Deep Learning

Table 2 is the data comparison of the “narrow road and dense network” planning mode design. The upper part and the lower part of the chart are the comparison of the effect before the renovation and the postrenovation effect of the optimization design research of street public space layout based on the Internet of Things and deep learning. From Table 2, it can be seen that, before the renovation, the roads in the street space layout have only a few small roads. After the renovation based on the Internet of Things and deep learning algorithms, the street space design has become a narrow road and dense network model. After the renovation, it is more attractive for leisure people to come for shopping and other commercial activities. The effect after the renovation is very good, especially for the development of the local business economy.

Table 3 is the optimized design of the street section. Using ArcGIS 10.1 software, the data model of the street spatial layout before the street reconstruction is abstracted. The original spatial layout of the street is only the width of the subway on both sides and the red line of the road in the middle. After the optimization design of street public space layout based on the Internet of Things and deep learning, the subway in the street space layout was replaced with three parts: (1) street pedestrian space, (2) riding space, and (3) building retreat. This transformation creates a commercial atmosphere, which is conducive to the development of local commercial economy.

Table 3 is the optimized design of the street section. Using ArcGIS 10.1 software, the data model of the street spatial layout before the street reconstruction is abstracted. The original spatial layout of the street is only the width of the subway on both sides and the red line of the road in the middle. After the optimization design of street public space layout based on the Internet of Things and deep learning, the subway in the street space layout was replaced with three parts: (1) street pedestrian space, (2) riding space, and (3) building retreat. This transformation creates a commercial atmosphere, which is conducive to the development of local commercial economy.

Judging from the actual test of the two programs, according to statistics, the number of commercial mobile personnel after the reform has increased exponentially, and the local commercial economy is also growing every year. The optimization design research of street public space layout based on the Internet of Things and deep learning improves the visual effect of the street space layout and the commercial atmosphere of the street space, which is conducive to the prosperity and development of the local business economy. The optimization design of street public space layout based on the Internet of Things and deep learning has fully improved the design level of street space layout, which can greatly promote economic development.

### 3.3. Suggestions for Optimization of Street Public Space Layout Based on Internet of Things and Deep Learning

#### 3.3.1. Supported by the Internet of Things, Promote Smart Parking Management

The countermeasures are adopted to solve the phenomenon of disorderly parking by classification. According to the pressure of the road network, scientifically assess the difference in parking demand in each area. Adopt the regulation mode of “supply on demand,” so that the limited parking resources can be used more optimally in terms of time and space, realize the off-peak utilization of parking resources, and, at the same time, ensure that the bus priority policy can be resolutely implemented. Penalties for passing private cars: using the Internet of Things system, the information sharing of parking spaces on the edge of the road is realized and integrated into the parking navigation. Realize the integration of functions such as parking space search and positioning, and alleviate the difficulties faced by residents when parking.

#### 3.3.2. Promote the Construction of Shared Streets

Improve the construction of street culture with the concept of sharing streets. Use various publicity tools to convey the idea that “the streets belong to all citizens” to citizens, especially roadside businesses. After the unified management of the billboards of the street merchants, the unification of the billboards in terms of size, color, and material is realized, thereby reducing the disturbance to pedestrians and effectively reducing the pollution to the environment. At the same time, it is necessary to reduce the behavior of laying advertisements in public areas as much as possible and make some board newspapers or paintings rich in literature and art and to promote the customs and people of turban to pedestrians. The publicity department of the community is responsible for regularly replacing and maintaining these contents, so as to provide a place for pedestrians to rest and create an artistic atmosphere in the public space of the street.

## 4. Conclusions

After entering the new century, most cities in China have realized the transformation from industrial cities to living cities. The concept of paying attention to urban livability has become increasingly popular among the people, and the public's use and emphasis on road public space is also increasing. Faced with this transformation, how to optimize the layout of urban streets has attracted the attention of many scholars. They proposed that the optimization of traffic plays a huge role in the transformation of urban space and the improvement of taste. In order to solve the problems of unbalanced street order, lack of quality, and loss of sense of security, many local governments have carried out design and planning to optimize road layout. Under the premise of not carrying out large-scale reconstruction of the existing urban road network, ways such as widening the roadway and channelizing the intersection are often used to improve the efficiency of urban traffic flow. Practice has proved that this method can only relieve traffic pressure in a short time and cannot achieve sustainable development. Therefore, this research is based on the optimization design of street public space layout based on the Internet of Things and deep learning and uses the index analysis model constructed by the Internet of Things and deep learning technology to explore an intermediate node, which can not only affect the urban traffic but also ensure the traffic flow safety. At the same time, it can also enhance the literary and unity of the street space. It is hoped that the problem of street public space layout will be completely solved, and the overall level of street space layout will be improved through rational planning of street public space.

## Figures and Tables

**Table 1:** Comparison of some data before and after the disorderly parking regulation.

	Average speed of motor vehicles (km/h)	Traffic accident rate
Park indiscriminately	16.5	46 times a week

After remediation	20.8	36 times a week

**Table 2:** Optimal planning diagram of charging pile location.

	Number of branches	Number of shoppers in the area
Original layout	4	127

Layout after optimization	9	196

**Table 3:** The optimized design of the street section.

	Subway and street
Original layout	(1) Subway
	(2) Main road

Layout after optimization	(1) Street pedestrian space
	(2) Arcade space
	(3) Building retreat

## Data Availability

The datasets used and/or analyzed during the current study are available from the corresponding author on reasonable request.
